# Prediction of coronary artery disease by a systemic atherosclerosis score index derived from whole-body MR angiography

**DOI:** 10.1186/1532-429X-11-36

**Published:** 2009-09-17

**Authors:** Stephanie Lehrke, Benjamin Egenlauf, Henning Steen, Dirk Lossnitzer, Grigorius Korosoglou, Constanze Merten, Boris T Ivandic, Evangelos Giannitsis, Hugo A Katus

**Affiliations:** 1 Abteilung Innere Medizin III, Medizinische Klinik, Universitätsklinikum Heidelberg, Heidelberg, Germany

## Abstract

**Background:**

Whole-body magnetic resonance angiography (WB-MRA) has shown its potential for the non-invasive assessment of nearly the entire arterial vasculature within one examination. Since the presence of extra-cardiac atherosclerosis is associated with an increased risk of coronary events, our goal was to establish the relationship between WB-MRA findings, including a systemic atherosclerosis score index, and the presence of significant coronary artery disease (CAD).

**Methods:**

WB-MRA was performed on a 1.5T scanner in 50 patients scheduled to undergo elective cardiac catheterization for suspected CAD. In each patient, 40 extra-cardiac vessel segments were evaluated and assigned scores according to their luminal narrowing. The atherosclerosis score index (ASI) was generated as the ratio of summed scores to analyzable segments.

**Results:**

ASI was higher in patients with significant (> 50% stenosis) CAD (n = 27) vs. patients without CAD (n = 22; 1.56 vs. 1.28, p = 0.004). ASI correlated with PROCAM (R = 0.57, p < 0.001) and Framingham (R = 0.36, p = 0.01) risk scores as estimates of the 10-year risk of coronary events. A ROC derived ASI of > 1.54 predicted significant CAD with a sensitivity of 59%, specificity of 86% and a positive predictive value of 84%. Logistic regression revealed ASI > 1.54 as the strongest independent predictor for CAD with a 11-fold increase in likelihood to suffer from significant coronary disease. On the contrary, while 15/27 (55%) of patients with CAD exhibited at least one extra-cardiac stenosis > 50%, only 3/22 (14%) of those patients without CAD did (p = 0.003). The likelihood for an extra-cardiac stenosis when CAD is present differed between vascular territories and ranged from 15% for a carotid stenosis to 44% for a stenosis in the lower extremities.

**Conclusion:**

This study provides important new evidence for the close association of extra-cardiac and coronary atherosclerosis. The novel findings that a WB-MRA derived systemic atherosclerosis score index is not only associated with established cardiovascular risk scores but is also predictive of significant CAD suggest its potential prognostic implications and underline the importance to screen for coronary disease in patients with extra-cardiac manifestations of atherosclerosis.

## Background

Atherosclerotic cardiovascular disease with its consequences of myocardial infarction and stroke is the leading cause of morbidity and mortality in the Western world [1]. Although the generalized nature of atherosclerosis has been recognized for decades [2,3], a systematic screening in patients presenting with a local manifestation of atherosclerosis, e.g. coronary artery disease (CAD), is not routinely performed. This is at least in part attributable to the lack of an established diagnostic approach as most of the major imaging techniques are limited by either their invasiveness (conventional angiography), their inability to assess large areas of the vascular tree at once (duplex sonography), their use of ionizing radiation (conventional angiography, computed tomography) and/or the administration of potentially toxic contrast agents.

In recent years, whole-body magnetic resonance angiography (WB-MRA) has evolved as a promising tool for the comprehensive assessment of nearly the entire arterial tree within one examination. With the advantages inherent to magnetic resonance imaging, i.e. lack of radiation exposure and use of well tolerated contrast agents, WB-MRA possesses the ideal characteristics not only for clinical assessment of the individual patient but also for epidemiological and interventional studies. In addition, there have been first reports on the potential of WB-MRA to create a single score reflecting the severity of systemic atherosclerotic burden [4-7]. In these studies, the respective "atherosclerosis score" was associated with traditional cardiovascular risk factors [4] and showed a correlation with the Framingham risk score [5]. This is in line with observations about the increased risk of coronary events in patients with extra-cardiac manifestations of atherosclerosis [8,9] and led the authors to postulate that a score of systemic atherosclerosis might be of value for cardiovascular risk assessment. These findings underline the importance to systematically establish the relationship between manifestations of extra-cardiac atherosclerosis as detected by WB-MRA and the presence and significance of coronary artery disease; however there is only scarce data on this question, limited mainly to the application of WB-MRA in patients with a history of coronary revascularization or prior myocardial infarction [4,10].

The aim of the present study was to assess the association between WB-MRA findings and the presence of significant coronary artery disease in patients undergoing elective cardiac catheterization. We further sought to evaluate a newly developed "atherosclerosis score index" as measure for systemic atherosclerotic burden with regard to its predictive value for the incidence of CAD and its correlation with established cardiovascular risk scores and other non-invasive surrogates for extra-cardiac atherosclerosis.

## Methods

### Study population

The study population consisted of 50 patients scheduled to undergo elective cardiac catheterization for suspected coronary artery disease at the Department of Cardiology, University Hospital Heidelberg, Germany. Patients were recruited from the cardiology outpatient clinic during February and March 2007. A total of 74 consecutive patients with the indication for elective cardiac catheterization for suspected CAD were screened. Pre-defined exclusion criteria for the study were [numbers of patients excluded based on each criteria]: known extra-cardiac atherosclerosis [n = 3], age < 18 years [n = 0], inability or unwillingness to give informed consent [n = 7], pregnancy [n = 0], renal failure with a glomerular filtration rate < 60 ml/min/1.73 m^2 ^[n = 5] and general contraindications to MRI such as presence of pacemakers [n = 0], implantable cardioverter defibrillators [n = 0], intracranial clips [n = 1], other foreign metallic objects [n = 3] or known severe claustrophobia [n = 5]. WB-MRA was performed several days before the date of the scheduled cardiac catheterization and ankle-brachial blood pressure index (ABI) was measured at the time of the MRI scan. PROCAM [11] and Framingham [12] scores were calculated for each patient as measures of the likelihood to suffer from a coronary event within the next 10 years. The study was performed in accordance to the Declaration of Helsinki. The ethics committee of the University of Heidelberg approved the study protocol and all patients gave their written informed consent.

### Whole-body magnetic resonance angiography

Whole-body MRA was performed on a 1.5T clinical scanner (Intera Achieva^®^, Philips Medical Systems, Best, The Netherlands) equipped with a table top extender using MobiTrak^® ^software (Philips Medical Systems). The system integrated quadrature body coil was used for signal transmission and reception. Patients were placed in a supine position feet-first. A survey scan with a time-of-flight sequence was performed for subsequent planning. MRA was acquired with a 3-dimensional T1 RF-spoiled gradient echo sequence in 4 slightly overlapping coronal data sets ("stations"). The first station included the adominal aorta and its branches, the second station started at the external iliac arteries and continued to the popliteal arteries, the third station contained the arteries of the lower leg. The fourth station consisted of the thoracic aorta and its supraaortic branches. Typical scan parameters were: TR 5 ms, TE 1.5 ms, flip angle 40°, FOV 451 mm, acquired voxel sizes 1.66 × 1.74 × 2 mm^3 ^(station 1), 1.41 × 1.71 × 1.8 mm^3 ^(station 2), 1.23 × 1.23 × 0.75 mm^3 ^(station 3) and 1.66 × 1.74 × 2 mm^3 ^(station 4). Reconstructed voxel size for all stations was 0.88 × 0.88 × 1 mm^3^. A breath-hold was used for station 1 only [5,6]. After acquisition of unenhanced images, 26 ml of gadopentetate dimeglumine (Magnevist^®^, Bayer Schering Pharma, Germany) were power injected (flow rate of 1.2 ml/sec for the first 13 ml, 0.4 ml/sec for the second 13 ml, followed by 25 ml saline with a flow rate of 0.4 ml/sec). Scanning of the first three stations was timed using real-time bolus monitoring (BolusTrak^®^, Philips Medical Systems), the image acquisition was started when the distal aorta and aortic bifurcation were clearly visible with contrast. After a wash-out period of 7 minutes, images of the fourth station were acquired in a similar fashion with the injection of 14 ml contrast media followed by 25 ml saline (flow rate for both 2 ml/sec). The scan was started when the ascending aorta was filled with contrast. For all stations, unenhanced images were subtracted from enhanced images. Maximum-intensity-projections were obtained from subtracted images in standard frontal and lateral projections. Multiplanar reconstructions were generated for image interpretation.

### Image analysis and calculation of the atherosclerosis score index

**Figure 1:**
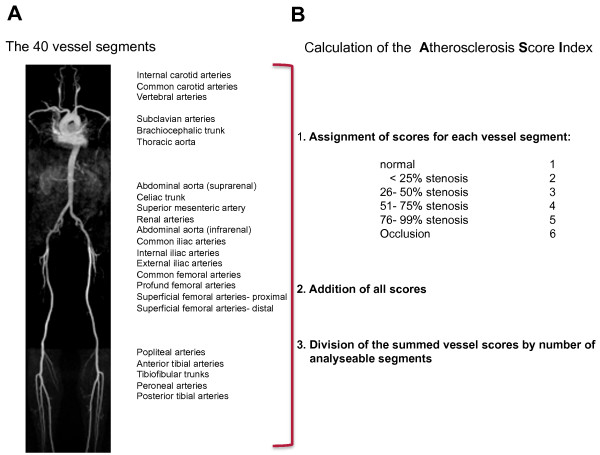
**Evaluation of WB-MRA**. Panel (**A**) shows an example for a fused whole body-MRA in a maximum intensity projection (MIP), the evaluated vessel segments are listed on the right. The ASI for each patient was calculated according to (**B**). The ASI in this example is 1.16.

Interpretation of WB-MRA data was performed on a commercially available work station (ViewForum^®^, Philips Medical Systems). For image analysis the arterial tree was divided into 40 segments ranging from the internal carotid artery to the arteries of the lower leg (Figure 1A). Using source images and multiplanar reconstructions all segments were graded with regard to their diagnostic quality (0 = non-diagnostic, 1 = poor image quality, diagnosis suspected, 2 = moderate quality, some blurring/artefacts but definite diagnosis possible 3 = good quality and 4 = excellent image quality) and their most severe stenosis (≤25%, 26- 50%, 51-75%, 76-99%, occlusion) by consensus reading of two experienced observers (S.L. and H.S.) blinded to the results of coronary angiography and other clinical data. Reasons for non-diagnostic quality were noted and included venous overlap, poor signal-to-noise ratio (SNR), motion artefacts and others (e.g. anatomic variants, orthopedic implants). All evaluable vessel segments received a score according to the severity of stenosis, ranging from 1 for a normal vessel to 6 for a total occlusion. The atherosclerosis score index (ASI) for each patient was calculated by dividing the sum of vessel scores by the number of analysed segments (Figure 1B).

### Coronary angiography

Cardiac catheterization was performed via the femoral or radial artery and coronary angiograms were obtained from all patients. Signficant coronary artery disease was defined as presence of vessel segments with > 50% luminal narrowing and was classified into 1- 2- and 3 vessel disease according to number of affected vessels. Coronary revascularization (percutaneous intervention or bypass grafting) was recommended at the discretion of the attending physician and either performed directly or at a later time point.

### Measurement of ankle-brachial blood pressure index (ABI)

The ABI was measured and calculated according to the recommendations of the American Heart Association [13]. A blood pressure cuff and a doppler ultrasonic sensor were used to determine systolic blood pressure in the brachial arteries and the posterior tibial and dorsalis pedis arteries. The ABI for each leg equaled the ratio of the higher of the two systolic pressures (posterior tibial or dorsalis pedis) and the average of the brachial pressures. In cases where there was a difference of > 10 mmHg between brachial artery pressures, the higher value was used. An ABI of < 0.9 was considered a pathological finding.

### Statistical analysis

Data were expressed as mean ± SEM or as median with interquartile range (IQR; 25-75th percentile) if not normally distributed. Differences between two groups were compared by Student's t-test or Mann-Whitney-U-Test for non-parametrical continuous variables. Categorical variables between groups were compared by chi-square test. The associations between ASI and Framingham and PROCAM scores were assessed using Pearson's correlation coefficient. ROC analysis was performed to determine the optimal ASI cut-off for diagnosis of significant CAD. Logistic regression analysis was carried out to provide the optimal model for prediction of the odds for significant CAD. For all analyses, a p-value of < 0.05 was regarded statistically significant. All statistical analyses were carried out using the Statistical Package for Social Science software version 16.0 (SPSS Inc., Chicago, USA).

## Results

Baseline patients' demographics and results of coronary angiography are displayed in Table 1. WB-MRA could be performed in all 50 patients without any adverse reactions to contrast media or major technical problems. One patient failed to keep her appointment for the scheduled cardiac catheterization.

**Table 1:** Baseline patient demographics and results from coronary angiography

Age (yrs)	61.7 ± 1.5
Age ≥ 70 yrs - n (%)	14 (28)
Male - n (%)	26 (52)
BMI (kg/m^2^)	27.6 ± 0.7
**Cardiovascular Risk**	
Diabetes - n (%)	16 (32)
Smoking - n (%)	14 (28)
Hypertension - n (%)	48 (96)
Hyperlipidemia - n (%)	36 (72)
Family history - n (%)	19 (38)
PROCAM Score	48.1 ± 1.9
Framingham Score	7.7 ± 0.53
**Coronary angiography**	
Significant CAD - n (%)	27/49 (55.1)
1 - vessel disease - n (%)	13/49 (26.5)
3 - vessel disease - n (%)	14/49 (28.6)
PCI - n (%)	14/49 (28.6)
CABG - n (%)	3/49 (6.1)

yrs: years; BMI: body mass index; CAD: coronary artery disease;

PCI: percutaneous coronary intervention; CABG: coronary artery bypass grafting

### WB-MRA-technical results and detection of stenoses

In 50 patients a total number of 1793/2000 (90%) vessel segments were evaluable. Excellent and good image qualities were found in 249 (14%) and 1235 (69%) segments respectively, while 270 (15%) segments showed moderate and only 39 (2%) poor diagnostic quality. Reasons for the non-diagnostic quality included motion artefacts (22/207), poor SNR (151/207), venous overlay (12/207) and others (22/207). Of the non-evaluable segments 52% were located in the abdominal area, while 18% were found in the supraaortic branches and 30% in the lower extremities.

A total of 27 stenoses > 50% were detected with a distribution among the different vessel territories as follows: 1. thoracic aorta/supraaortic branches n = 5 (19%); 2. abdominal aorta with visceral and pelvical branches n = 10 (37%); 3. lower extremities starting with the common femoral artery n = 12 (44%). A total of 18 patients (36%) were found to exhibit at least one luminal narrowing > 50%, among these, 6 patients had two ore more > 50% stenoses.

### The Atherosclerosis Score Index (ASI)

**Figure 2:**
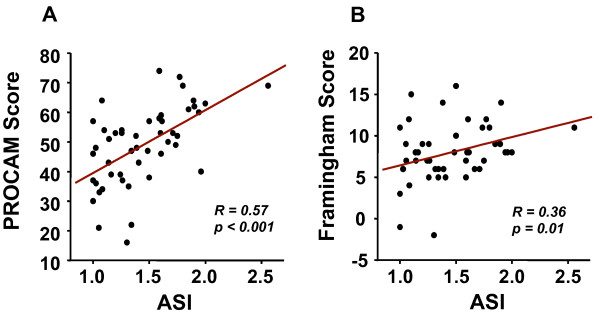
**Association between ASI and cardiovascular risk scores**. ASI showed a good correlation with PROCAM (**A**) and Framingham (**B**) scores, suggesting its possible prognostic value.

The mean ASI among the study cohort was 1.43 ± 0.05, ranging from 1 to 2.56. A higher ASI was not associated with older age (> 70 years), gender or traditional cardiovascular risk factors. However, ASI was markedly higher in patients with an ABI < 0.9 (Table 2). Significant correlations were found between the ASI and PROCAM as well as Framingham risk scores (Figure 2).

**Table 2:** ASI according to age, gender, traditional risk factors and ABI

	**ASI**		**ASI**	**p-value**
male gender	1.47 ± 0.08	female gender	1.39 ± 0.06	ns
diabetes	1.49 ± 0.1	no diabetes	1.4 ± 0.05	ns
age ≥ 70 yrs	1.58 ± 0.11	age < 70 yrs	1.37 ± 0.05	ns
smoker	1.43 ± 0.07	non-smoker	1.43 ± 0.06	ns
hypertension	1.44 ± 0.05	no hypertension	1.17 ± 0.03	ns
hyperlipidemia	1.44 ± 0.06	no hyperlipidemia	1.4 ± 0.09	ns
ABI = 0.9	1.76 ± 0.12	ABI > 0.9	1.36 ± 0.05	0.01

ASI: atherosclerosis score index; yrs: years; ABI: ankle-brachial blood pressure index

### Association between coronary artery disease and WB-MRA findings

**Figure 3:**
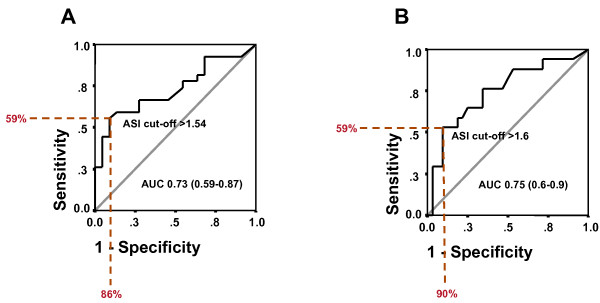
**Prediction of CAD and coronary revascularization by ROC derived ASI cut-offs**. While an ASI > 1.54 proved to be the optimal discriminator for presence of significant coronary artery disease (**A**), the need for coronary revascularization was best predicted by a slightly higher ASI cut-off of 1.6 (**B**). For both, the sensitivities were 59%, the specificity for prediction of CAD was 86% (positive predictive value 84%) and the specificity for precition of coronary revascularization was 90% (positive predictive value 63%).

**Figure 4:**
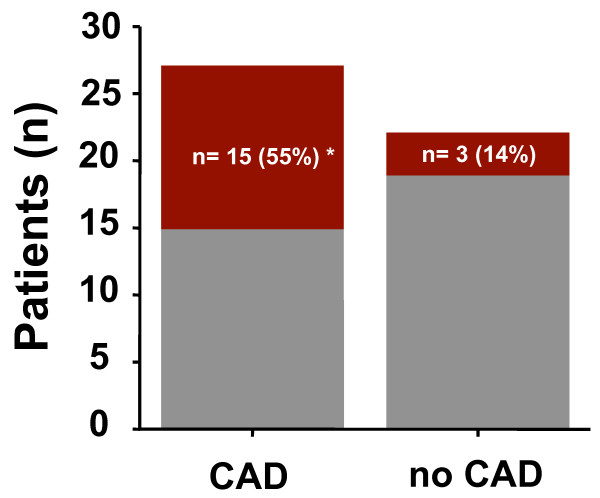
**Association of higher-grade extra-cardiac stenoses with significant CAD**. While 55% of patients with CAD had at least one higher-grade (> 50%) extra-cardiac stenosis, only 14% of patients without CAD did (* p = 0.003). On the contrary, 15/18 (83%) patients who showed a higher-grade extra-cardiac stenosis also suffered from significant coronary artery disease. (Grey columns: patients without higher-grade extra-cardiac stenoses; red columns: patients with higher-grade extra-cardiac stenoses).

Patients with significant coronary artery disease (n = 27) exhibited a higher ASI compared to patients without significant coronary disease (n = 22; 1.56 vs. 1.28, p = 0.004). Furthermore, ASI was markedly elevated in patients undergoing coronary revascularization (1.6 vs. 1.34, p = 0.01). ROC analysis revealed an ASI cut-off of >1.54 as the optimal discriminator for the presence of significant CAD with a sensitivity of 59%, a specificity of 86% and a positive predictive value of 84%. Likewise, a cut-off for severe CAD necessitating coronary revascularization of >1.6 could be determined with a sensitivity of 59%, a specificity of 90% and a positive predictive value of 63% (Figure 3). Significant CAD was associated with the presence of higher grade extra-cardiac stenoses - while 15/27 (55%) of patients with CAD exhibited at least one extra-cardiac stenosis > 50%, only 3/22 (14%) of those patients without CAD did (p = 0.003, Figure 4). The incidence of a significant extra-cardiac stenosis when CAD was present differed between vessel territories. While 4/27 (15%) patients with CAD suffered from carotid artery stenoses, higher grade stenoses in the abdominal/pelvical regions were present in 7/27 (26%) patients and a total of 12/27 (44%) patients had concomitant stenoses of the arteries in the lower extremities.

**Figure 5:**
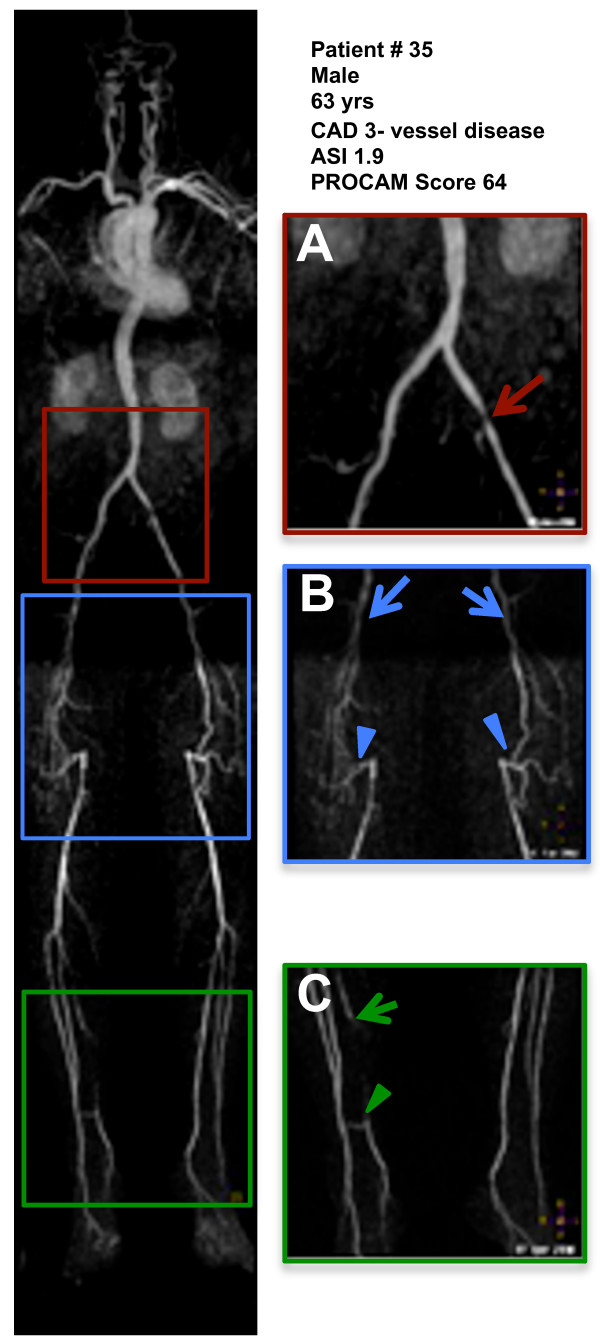
**Patient example**. WB-MRA of a 63 year old male with stenosis of the left common iliac artery (**A**, red arrow), occlusion of both superficial femoral arteries (**B**, blue arrows) with collateralization by the profund femoral arteries (blue arrowheads) and occlusion of the right posterior tibial artery (**C**, green arrow) with collaterals from the anterior tibial artery (**C**, green arrowhead). The patient did not complain about claudicatio at the time of the exam. Based on the results of WB-MRA regular angiological follow-ups were initiated. About 1 year after the MRA angioplasty of the left iliac artery was performed. Coronary angiography revealed 3-vessel disease and the patient underwent PCI of the left anterior descending artery. ASI and PROCAM Score were high.

The presence of a stenosis > 50% was slightly less powerful compared to the ASI discriminator in predicting the presence of CAD with a sensitivity of 56% and a specificity of 86% (positive predictive value 83%). Figure 5 displays an example of a patient with a high ASI and 3-vessel coronary disease.

Together with other variables associated with CAD in univariate analysis, the ASI cut-off of 1.54 was used to build logistic regression models predicting the odds of significant CAD. Table 3 displays the best-fit model. Among the variables entered (age > 70 yrs, male gender, ABI < 0.9, PROCAM score), the odds ratio was statistically different from 1 for ASI > 1.54 only. In the specific model, an ASI > 1.54 was associated with a 11-fold increase in likelihood to suffer from significant CAD. When entered into logistic regression models as a continuous variable ASI did not retain its independent predictive value.

**Table 3:** Logistic regression for prediction of significant coronary artery disease

	**B**	**S.E.**	**p-value**	**OR**	**95% CI**
PROCAM Score (per point)	-0.02	0.05	0.69	0.98	0.89-1.08
Age (per year)	-0.01	0.04	0.9	1	0.93-1.07
Male gender	1.21	0.7	0.08	3.37	0.85-13.3
ABI < 0.9	0.53	1.12	0.64	1.7	0.19-15.33
ASI > 1.54	2.4	0.92	0.009	10.99	1.8-67.1

ABI: ankle-brachial blood pressure index; ASI: atherosclerosis score index

## Discussion

The present study confirms the close relationship between extra-cardiac atherosclerosis as assessed by whole-body magnetic resonance angiography and the presence of coronary artery disease. The major new finding of this study is the observation that significant coronary artery disease can be predicted from a WB-MRA derived atherosclerosis score index reflecting systemic atherosclerosis.

The systemic nature of atherosclerosis has been recognized for decades [2,3]. Despite this fact, the clinician's attention is usually focused on the local manifestations of atherosclerosis, e.g. coronary artery disease, peripheral arterial occlusive disease or cerebrovascular disease, which are responsible for the patients' symptoms and thus the reason why medical attention is sought. While the diagnosis of any form of atherosclerosis will prompt the initiation of tertiary prevention including medical therapy and life style modification affecting the whole body, more specific treatment measures may be necessary for other locations, e.g. high grade internal carotid artery or renal artery stenosis. It is therefore desirable to perform a systemic screening in any patient presenting with a manifestation of atherosclerosis.

In recent years whole-body MR angiography has evolved as a promising non-invasive technique for the assessment of nearly the entire arterial system in one examination. The feasibility of this technique on routine clinical 1.5 T MRI scanners has been widely established [14-17] and in terms of diagnostic accuracy excellent agreements with digital subtraction angiography as the gold standard could be shown [18-20]. As a multi-station bolus-chase technique, WB-MRA possesses the inherent difficulty of optimal timing of image acquisition to keep up with the contrast bolus. This is especially challenging with regard to smaller arteries in the calf region where a higher spatial resolution is desirable but leads to longer acquisition times which in turn increase the venous contamination and thus hamper the diagnostic accessibility. One solution to this problem is the application of a sub-systolic venous compression which can be achieved by the application of a blood pressure cuff to the thigh region. Venous thigh compression was shown in WB-MRA to improve SNR and CNR, to ameliorate venous contamination and to lead to a higher sensivity and specificity for the detection of stenoses and more congruency in grading of stenoses which had been found by DSA [21,22]. Importantly, venous compression changes contrast dynamics [23] and contrast flow rates need to be adapted [24].

Apart from subsystolic venous compression, which mainly aims at improving image quality in the distal portions of the lower extremities, an established method to increase signal, decrease scanning times and/or improve spatial resolution in MRA is the application of parallel imaging techniques with the use of multi-channel receiver coils. These have already successfully been applied to MR whole body angiography [16,19,25]. While this approach leads to an improvement in image quality, it requires special hardware. In this study, we aimed to apply an easy- to - use protocol by using the system integrated quadrature body coil and refrained from using venous thigh compression.

With regard to clinical applications, the potential of WB-MRA to detect unknown arterial disease was demonstrated in different patient cohorts such as diabetics [4], patients with a history of coronary revascularization or myocardial infarction [10] and a large population of elderly Swedish citizen [6]. The impact of the diagnosis of additional stenoses on the clinical management in patients with peripheral arterial occlusive disease was demonstrated by Goyen et al [26].

In addition to the detection of individual high-grade stenotic lesions with consequences for the treatment strategy of the patient, the systemic nature of atherosclerosis makes it tempting to establish a marker reflecting the total atherosclerotic burden to be used for epidemiological and interventional studies. With this in mind we used the information from WB-MRA to design an atherosclerosis score index, which is similar to the one recently published by two other groups [4,5] in selected patient populations.

The good correlation of the vessel scores with cardiovascular risk factors such as smoking, systolic blood pressure, male gender and the Framingham Risk Score [4], the latter being confirmed in our own findings, makes it tempting to speculate that this score may be of prognostic significance. This idea is supported by earlier reports in which non-invasive measures of extracoronary atherosclerosis such as ankle-brachial-index and intima-media thickness were shown to be associated with an increased risk of cardiovascular events [8,9], and that on a morphological level coronary artery disease as detected by computed tomography is associated with extra-cardiac atherosclerosis [10,27,28].

The strong association between coronary and extra-coronary atherosclerosis is confirmed by our own results. The presence of significant coronary artery disease was associated with a pronounced systemic atherosclerotic burden as reflected by a markedly increased atherosclerosis score index. In our study population of patients undergoing cardiac catheterization for clinically suspected coronary disease a fairly large percentage (45%) did not exhibit significant (i.e. > 50% stenosis) CAD. This led us to the question whether ASI can help to discriminate between patients with vs. without significant coronary disease. In fact, a ROC analysis derived ASI cut-off of > 1.54 had a sensitivity of 59%, a specificity of 86% and a positive predictive value for CAD of 84%. When entered into logistic regression models an ASI > 1.54 remained the strongest independent predictor for CAD. In addition to the somewhat complex ASI the presence of a single > 50% extra-cardiac stenosis also showed a relatively good positive predictive value for significant CAD (83%), was however not as strong a predictor when entered into logistic regression models. Interestingly, despite the good specificity, the use of WB-MRA to screen for CAD to potentially avoid cardiac catheterization can not be recommended at this time as peripheral atherosclerosis has a low sensitivity to detect CAD with only 55% of patients with CAD showing higher-grade extra-cardiac stenoses. This prevalence is comparable with the WB-MRA findings reported in patients with known coronary artery disease by Ladd et al [10] and in line with earlier reports assessing extra-cardiac atherosclerosis in patients with CAD by other non-invasive methods [29]. These observations in conjunction with the finding that the percentage of CAD patients exhibiting other atherosclerotic manifestations increases with advanced age [29], support the theory that coronary artery disease is an early manifestation of atherosclerosis. The use of WB-MRA to screen for CAD and potentially avoid cardiac catheterization can therefore not be recommended.

Despite the promising reports about a WB-MRA derived atherosclerosis score by us and others [4-7], the technique is limited by the fact that it is a "luminography" and does not image plaque burden or plaque vulnerability. With new technical developments leading to higher spatial resolution it may be feasible in the future to combine WB-MRA with plaque imaging in a reasonable amount of time. Importantly, if a WB-MRA derived atherosclerosis scoring system is to become an established method, it needs to be standardized to allow reproducibility. The three scoring systems, including ours, which have been developed almost simultaneously, differ in their approaches. The total atherosclerosis score by Hansen et al. is based on the evaluation of 26 vessel segments divided into 5 territories to allow for a weighting of different stenosis localizations over others (e.g. a carotid artery stenosis contributes more to the total score than a tibial artery stenosis) [5]. Weckbach et al used a system to calculate their atherosclerosis score similar to ours, however evaluated only 22 vessel segments [4]. Our own ASI is characterized by grading of a large number of vessel segments. Interestingly, although all three scores are not directly comparable, the published results complement each other very well and underline the potential of this new method.

One limitation of our study is that not all vessel segments were evaluable. Diagnostic quality was diminished especially in the abdominal vessel regions due to low SNR. It is thus possible that renal artery stenoses were missed. The association with renal artery stenoses and CAD has been well recognized [30], it is thus likely that an improvement of image quality with the detection of more renal artery stenoses will further strengthen our findings of the close association between extra-cardiac and coronary atherosclerosis. We believe that limitations in image quality can be overcome by new technical developments aiming to improve spatial resolution and signal-to-noise ratio such as imaging at 3 T [25,31], the use of multi-channel receiver and body-surface coils with parallel imaging techniques [16,19,25], subsystolic venous compression [21-24] and possibly the application of blood pool contrast agents [32].

## Conclusion

Our findings support the concept of a WB-MRA derived atherosclerosis score index as marker for systemic atherosclerotic burden. The close relationship between the atherosclerosis score index and the presence of significant coronary artery disease and its correlation with traditional established risk scores suggest that the score may be of prognostic significance and underlines the importance to screen for coronary disease in patients with extra-cardiac stenoses. We further postulate that the ASI will be a valuable tool for longitudinal studies of atherosclerosis' pathophysiology and progression.

## Competing interests

The authors declare that they have no competing interests.

## Authors' contributions

EG and HAK conceived the study and participated in the design and coordination of the study. SL and HS carried out the analyses of the whole-body magnetic resonance angiographies and drafted the manuscript. DL and SL performed the statistical analyses. BTI contributed to the conception of the study and helped with the statistical analyses. CM and GK participated in the design of the study and critically revised the manuscript. BE recruited patients, collected and tabulated all clinical data and helped to draft the manuscript. All authors read and approved the final manuscript.
